# Cell Lysis in *S*. *pombe ura4* Mutants Is Suppressed by Loss of Functional Pub1, Which Regulates the Uracil Transporter Fur4

**DOI:** 10.1371/journal.pone.0141796

**Published:** 2015-11-04

**Authors:** Kohei Nishino, Misaki Kushima, Yuzy Matsuo, Yasuhiro Matsuo, Makoto Kawamukai

**Affiliations:** Department of Life Science and Biotechnology, Faculty of Life and Environmental Science, Shimane University, Matsue, Japan; Kinki University School of Pharmaceutical Sciences, JAPAN

## Abstract

*Schizosaccharomyces pombe* Δ*ura4* cells lyse when grown on YPD medium. A *S*. *pombe* non-essential gene deletion library was screened to determine suppressors of the lysis phenotype. Deletion of the *pub1* gene, which encoded E3 ubiquitin ligase, strongly suppressed cell lysis in Δ*ura4* cells. The Δ*pub1* cells displayed high sensitivity to 5-fluorouracil, a toxic analog of uracil, and this sensitivity was suppressed by deletion of *fur4*, which encoded a uracil transporter. Fur4 localized primarily to the Golgi apparatus and vacuoles in wild-type cells, but localization was predominantly at the plasma membrane in Δ*pub1* cells. Fur4 was necessary for the utilization of extracellular uracil, cytosine, or UMP. Uracil uptake activity increased in the Δ*pub1* strain in a Fur4-dependent manner. In addition, uracil starvation was critical for induction of cell lysis of Δ*ura4* strains and uracil supplementation suppressed lysis. In summary, the increased uracil uptake ability of Δ*pub1* cells, where Fur4 was predominantly localized to the plasma membrane, resulted in suppression of cell lysis in the Δ*ura4* background.

## Introduction

The fission yeast *Schizosaccharomyces pombe* is a popular model organism due to its amenability to genetic, molecular, and cell biological analysis. *S*. *pombe* has been used for the study of numerous biological processes such as the cell cycle, signal transduction, cell morphogenesis, chromatin structure, and metabolism [1–4]. The *S*. *pombe ura4* gene is particularly valuable as a marker for gene disruption or as a reporter to monitor gene expression at a locus of insertion. The *ura4* gene encodes orotidine-5-monophosphate (OMP) decarboxylase in the *de novo* uridine-5-monophosphate (UMP) synthesis pathway [5,6]. Previously, we reported that *S*. *pombe ura4* mutant cells underwent lysis when grown in a medium containing polypeptone, such as YPD [7]. YPD is widely used for growth of *Saccharomyces cerevisiae*. However, YPD is not commonly used for *S*. *pombe*, because cultivated lines often exhibit unexpected and unwanted alterations in their phenotypes of interest. This is particularly apparent in *S*. *pombe ura4* mutants; however, similar effects have not been observed in mutants of the *S*. *cerevisiae* counterpart, *URA3*.

Cell lysis is observed specifically in *S*. *pombe ura4* mutants and is not observed in other uracil auxotrophs (*ura1*, *ura2*, *ura3*, and *ura5* mutants) [7]. A precursor molecule, OMP, accumulates substantially in *ura4* mutants, and this might act as a trigger for cell lysis [7]. Cell lysis is suppressed by the addition of uracil. This suppresses the accumulation of OMP, probably because uracil inhibits the *de novo* UMP pathway at a point upstream of the Ura4 reaction. Addition of sorbitol to growth media maintains high osmolarity and, consequently, also suppresses cell lysis.

To further understand the mechanisms underlying cell lysis in *S*. *pombe ura4* mutants, we performed a suppressor screen using a *S*. *pombe* gene deletion library obtained from Bioneer Corp. [8]. Screening of 3,400 disruptants revealed several putative suppressors including *Δpub1*, which strongly suppressed cell lysis in *ura4* mutants. The *pub1* gene encodes a HECT-type E3 ubiqutin ligase [9]. Pub1 is associated with low pH tolerance, regulation of leucine uptake in response to NH^4+^, and the cell cycle [10]. Pub1 is also required for membrane localization of some membrane proteins, including the amino acid and peptide transporters Aat1, Cat1, and Ptr2, and the GPI anchored protein Ecm33 [11–14]. Ubiquitination of Aat1 or Cat1 alters their localization.

In this study, we screened a *S*. *pombe* mutant library for suppression of cell lysis in *ura4* mutants and analyzed the mechanism underlying *pub1* suppression. Our results showed that Pub1 altered the localization of the uracil transporter Fur4 from the Golgi locus and vacuoles to the plasma membrane. When Fur4 was predominantly localized at the membrane, uracil uptake increased and cell lysis was suppressed. A novel regulatory mechanism regarding Fur4 and its relationship with cell lysis is proposed.

## Materials and Methods

### Strains and media

The *S*. *pombe* strains used in this study are listed in Table 1. Standard yeast culture media and genetic manipulations were used [15]. *S*. *pombe* strains were grown in complete YES medium (0.5% yeast extract (Oxoid Ltd.), 3% glucose, and 225 mg/l each of adenine, leucine, uracil, histidine, and lysine hydrochloride), in YE (1% yeast extract and 2% glucose), in YPD medium (1% yeast extract, 2% glucose, and 2% polypeptone (Nihon Pharmaceuticals Co. Ltd.)), or in EMM medium (0.3% potassium hydrogen phthalate, 0.56% sodium phosphate, 0.5% ammonium chloride, 2% glucose, vitamins, minerals, and salts) [15]. EMM(-N) medium lacked NH_4_Cl. The appropriate auxotrophic supplements were added as necessary (225 mg/l of leucine and/or uracil) to EMM or EMM(-N). 5-Fluorouracil (5-FU) was added to a final concentration of 1, 3, 5, 50, 200, or 500 μM.

**Table 1:** Strains used in this study.

Strain	Genotype	Reference
L972	*h* ^−^	Lab stock
PR109	*h* ^−^ *ura4-D18 leu1-32*	Lab stock
UMP31	*h* ^−^ *ura4*::*kanMX6*	[7]
KNP16	*h* ^−^ *fur4*::*hphMX6*	This study
KNP25	*h* ^−^ *pub1*::*natMX6*	This study
KNP27	*h* ^-^ *ura4*::*kanMX6 fur4*::*hphMX6*	This study
KNP32	*h* ^-^ *ura4*::*kanMX6 pub1*::*natMX6*	This study
KNP38	*h* ^-^ *ura4*::*kanMx6 fur4*::*hphMX6 pub1*::*natMX6*	This study
KNP63	*h* ^−^ *ura4-D18 leu1-32 pub1*::*natMX6*	This study
KNP76	*h* ^-^ *fur4-GFP-hphMX*	This study
KNP83	*h* ^-^ *pub1*::*natMX6 fur4-GFP-hphMX6*	This study
KNP87	*h* ^-^ *pub1*::*natMX6 fur4*::*hphMX6*	This study
KNP95	*h* ^-^ *ura4*::*kanMX6 fur4-GFP-hphMX6*	This study

### Construction of expression plasmid

To construct the plasmid expressing Fur4-GFP, the *fur4-GFP* fusion gene was amplified by PCR from the genomic DNA of KNP76 using a set of primers of fur4P-NdeI and pFA6a-d-SalISmaIR or pFA6a-d-SalISmaIF and 13MYC-SR (S1 Table). Two pieces of the fragment were combined by PCR using primers of fur4P-NdeI and 13MYC-SR. After digesting with NdeI and SmaI, the fragment was cloned into the same sites of pREP42. The sequence of pREP42-Fur4-GFP plasmid was confirmed by sequence analysis.

### Gene disruption

Chromosomal genes were disrupted using PCR generated fragments [16]. The 1.5 kb *kanMX6*, 1.7 kb *hphMX6*, and 1.2 kb *natMX6* modules were amplified with flanking homology sequences corresponding to the target genes [17]. Correct disruption of the gene of interest was verified by colony PCR using appropriate primers [18]. The GFP C-terminal tagged *fur4* gene was also generated using PCR. Tag incorporation was confirmed by colony PCR and by immunoblotting with specific antibodies. The primers used in this study are listed in S1 Table.

### Screening for extragenic suppressors of *ura4-D18*

For primary screening of the gene deletion library (Ver. 4) from Bioneer Corp., cells were pre-cultured on YES medium and subsequently spotted onto YPD medium for incubation at 30°C for 3 days. For the alkaline phosphatase assay, plates were overlaid with 1% agar containing 0.05 M glycine-NaOH (pH 9.8) and 2.5 mg/ml of 5-bromo-4-chloro-3-indorylphosphate (BCIP), and incubated for 10, 30, and 60 min. After this primary screen, putative suppressor mutants were re-suspended in water to a density of 4 × 10^6^ cells/ml. The cell suspensions were serially diluted (1:10), spotted on YPD plates, and incubated for 3 days at 30°C. A further BCIP assay was also performed. Putative suppressor mutant cells were also observed by microscopy.

### Spot assay

Cells were grown on YES plates for 3 days at 30°C and were then re-suspended in water to a density of 2 × 10^6^ cells/ml. Cell suspensions were serially diluted (1:10), spotted onto YES, YPD, or EMM plates, and incubated for 4 days at 30°C. The alkaline phosphatase assay was performed as described above.

### Fluorescence microscopy of GFP and RFP

Fur4-GFP tagged *S*. *pombe* cells were pre-grown to log-phase in YES medium. Cells were then washed twice in MilliQ water, transferred to YES, YPD, EMM, EMMU (EMM+Uracil), EMM(-N), or EMMU(-N) media, and grown at 30°C for 12 h. Fur4-GFP or Gms1-RFP fluorescence of living cells was observed using a BX51 fluorescent microscope (Olympus Corp.) connected to a digital camera DP70 (Olympus Corp.). We used imageJ software (NIH, Bethesda, MD) to merge two images. Vacuolar membrane was visualized with FM4-64. Cells were incubated with 8μM FM4-64 for 1h at 30°C and then washed twice with dH_2_O to remove free FM4-64.

### Detection of ubiquitinated Fur4 protein

Polyubiquitination analysis was performed as described previously [19]. For detection of the polyubiquitinated Fur4 protein, wild-type or mutant *S*. *pombe* cells were transformed with plasmid pREP42 or pREP42-Fur4-GFP and pREP1 or pREP1-6His-AtUbiquitn (provided by Dr. H. Seino), which contained a gene encoding 6×His tagged ubiquitin under the *nmt1* promoter. Cells were cultured at 30°C in EMM medium for 18 h. Cells were then harvested by centrifugation and washed once with ice-cold stop buffer (150 mM NaCl, 50 mM NaF, 10 mM EDTA, and 1 mM NaN_3_, pH 8.0). Whole cell extracts were prepared in denaturing Buffer U (8 M Urea, 100 mM sodium phosphate, and 50 mM Tris-HCl, pH 8.0) and incubated for 1 h with Ni^2^-NTA agarose beads (Qiagen Corp.) at room temperature. The beads were then washed four times with Buffer U. Precipitated proteins were separated by SDS-PAGE and transferred to a nitrocellulose membrane. Membranes were immunoblotted with anti-GFP antibody (Roche Ltd.) at a 1/1,000 dilution. Detection of proteins was performed using the ECL system (GE Healthcare UK, Ltd.). Membranes were also probed using an anti-ubiquitin antibody (U5379; Sigma-Aldrich Co.), at a 1/100 dilution, as a loading control.

### Measurement of uracil in medium by LC-MS

Exponentially growing cells in YES medium were transferred to fresh YPD medium at time point 0 and cultured at 30°C. Initial cell concentrations in YPD were 5 × 10^5^, 5 × 10^6^, or 5 × 10^7^ cells/ml. Culture aliquots were taken at each desired time point, and after removal of cells by centrifugation and addition of acetonitrile to a final concentration of 50%, supernatants were stored at 4°C. LC-MS data were obtained using a MassLynx system (Waters) coupled to a Xexo-TQ mass spectrometer (Waters). LC separation was performed on an ACQUITY UPLC BEH Amide column (Merck SeQuant; 2.1×100 mm, 1.7 μm particle size). Buffer A (acetonitrile + 0.1% formic acid) and buffer B (H_2_O + 0.1% formic acid) were used as the mobile phase, with gradient elution from 80% A (20% B) to 62% A (38% B) in 3.5 min at a 0.4 ml/min flow rate. The initial conditions were restored after 4 min and maintained for 7 min at a flow rate of 0.4 ml/min. Uracil was detected using MRM mode (ESI (+) 113>96)

### Uracil transport assay

The efficiency of cellular uracil uptake was assessed as described previously [20,21]. Cells were cultivated in YES medium at approximate densities of 4 × 10^6^–1 × 10^7^ cells/ml. Cells were collected, and 2 × 10^7^ cells were washed twice with sterile water, suspended in 4 μM [U-^14^C]uracil (Moravek Biochemicals Inc.) for 1 min at room temperature, then quickly filtered using an Omnipore membrane filter (Millipore Corp., 47 mm diameter and 1 μm pore size). Filters were washed twice with sterile water, and radioactivity was counted using a Beckmann LS6000TA scintillation counter.

## Results

### Screen for suppressors of YPD-induced cell lysis in *ura4* mutants

**Fig 1:**
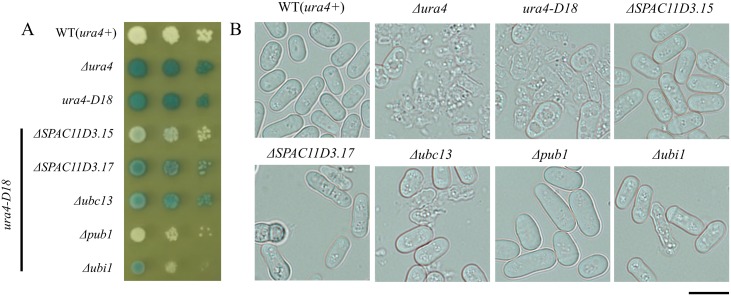
Deletions suppressing cell lysis in Δ*ura4* strains grown on YPD medium. (A) L972 (*ura4* ^+^), UMP31 (Δ*ura4*), KT35 (*ura4*-*D18*), and indicated deletion mutants (Bioneer deletion library ver4.0) were grown for 12 h, then spotted onto YPD medium and incubated for 3 days at 30°C. For the alkaline phosphatase assay, YPD plates were overlaid with a phosphatase assay solution containing 50 mM glycine-NaOH (pH 9.8), 1% agar, and 2.5 mg/ml of BCIP for 30 min. (B) Cells were also observed by microscopy after 3 days of growth on YPD. Five examples of mutants exhibiting different levels of lysis suppression are shown. Bar: 10 μm.

Previously, we showed that cell lysis was induced by polypeptone in *S*. *pombe ura4* mutants [7]. Approximately 90% of cells burst when *S*. *pombe ura4* mutants were grown in YPD medium until the stationary phase. To further understand the mechanisms governing this cell lysis, we screened a 3,400 non-essential gene deletion library (Bioneer Corp.) for lysis suppressors. The deletion library contained *leu1-32*, *ura4-D18*, and *ade6-M210* (or *ade6-M216*) mutations as well as individually disrupted genes (replaced with a kanamycin resistance gene). It was expected that this library would include some strains in which polypeptone-induced cell lysis was suppressed. The presence of the *ura4-D18* mutation in the library facilitated screening. All 3,400 strains were pre-cultured on YES medium, and then spotted onto YPD medium and incubated at 30°C for 3 days. Severity of cell lysis was measured using a BCIP assay. In the assay, lysed cells turned blue as a result of alkaline phosphatase release from burst cells. Unlysed cells remained white (Fig 1A). Of the 3,400 strains, 125 putative suppressor mutants were identified in the primary screening that were less blue than the parental strain. For secondary screening, the 125 putative suppressor strains were grown in YES medium, appropriately diluted, and spotted on YPD medium before verification of cell lysis intensity by microscopic examination (Fig 1B). The secondary screening identified 123 suppressor strains, some of which are shown in Fig 1. The *Δura1*, *Δura2*, *Δura3*, and *Δura5* strains, which we previously identified as *Δura4* lysis suppressors [7], were identified, lending confidence to the validity of our screening approach. Of the remaining suppressor strains, we observed that lysis was particularly strongly suppressed in the *Δpub1* strain, and we investigated this further. The *pub1* gene encodes a HECT-type E3 ubiqutin ligase, which is homologous to *S*. *cerevisiae RSP5* [22]. The *ΔSPAC11D3*.*15* strain was selected as a good suppressor (Fig 1A), but the later analysis turned out that this strain contains an additional mutation that affects cell lysis.

### Pub1 is involved in uracil uptake

**Fig 2:**
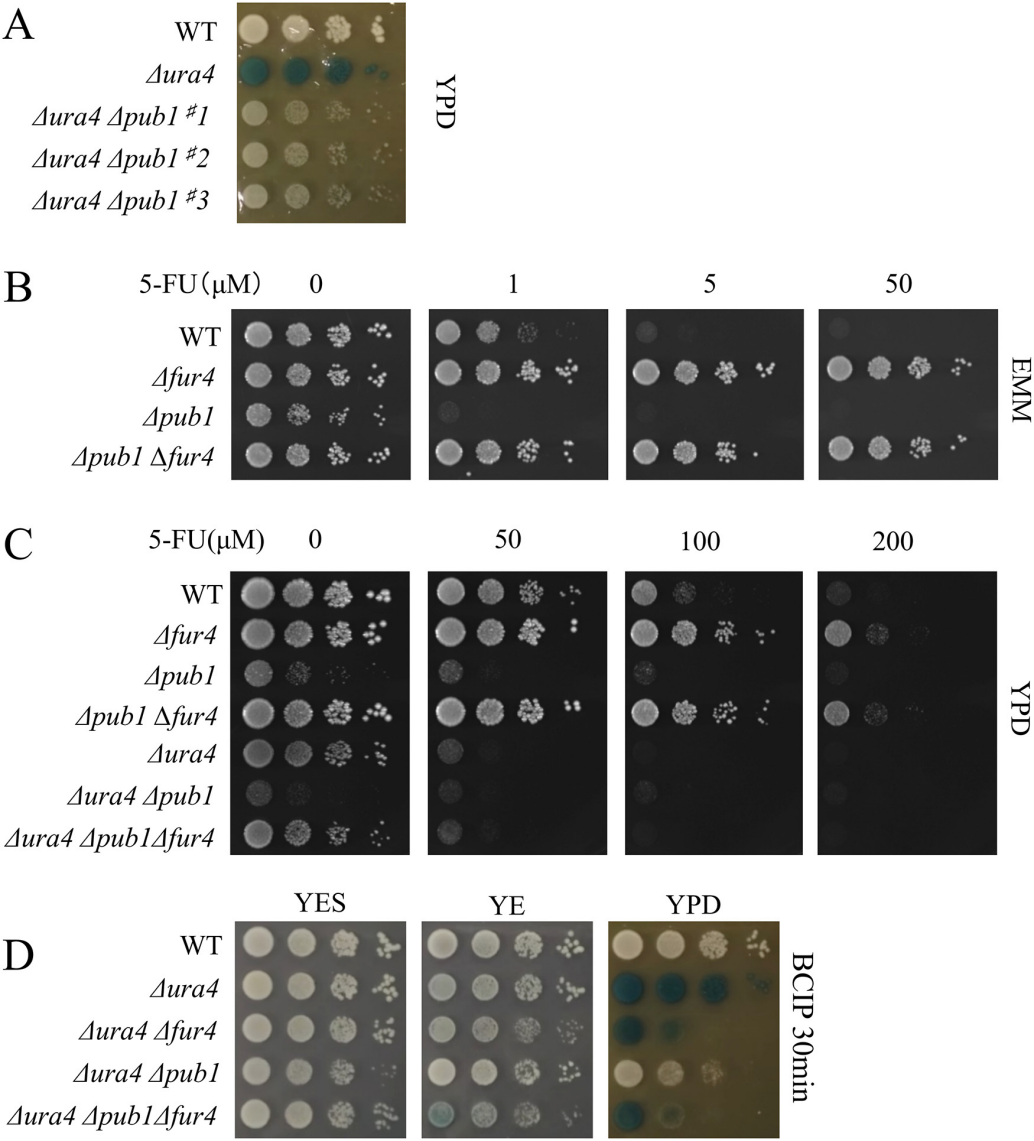
Suppression of cell lysis and 5-FU sensitivity in Δ*pub1* cells. (A) L972, UMP31 (Δ*ura4*), and KNP32 (Δ*ura4* Δ*pub1*) were grown for 12 h in YES liquid medium. These cells were spotted on YPD or YPD+Ade+Leu medium and incubated for 3 days. For the alkaline phosphatase assay, BCIP was used as described in Fig 1. (B and C) L972 (*ura4* ^+^), KNP16 (Δ*fur4*), KNP25 (Δ*pub1*), KNP87 (Δ*pub1*Δ*fur4*), UMP31 (Δ*ura4*), KNP32 (Δ*ura4* Δ*pub1*), and KNP38 (Δ*ura4* Δ*pub1* Δ*fur4*) cells were grown in YES liquid medium for 12 h. The indicated cells were spotted on EMM (B) or YPD (C) in the presence or absence of 5-FU (1, 5, 50, 100, or 200 μM) and then incubated at 30°C for 3 days. (D) L972 (*ura4* ^+^), UMP31 (Δ*ura4*), KNP32 (Δ*ura4* Δ*pub1*), and KNP38 (Δ*ura4* Δ*pub1* Δ*fur4*) cells were grown in YES liquid medium for 12 h. These cells were spotted on YES, YE, and YPD plates and incubated at 30°C for 3 days. For the alkaline phosphatase assay, BCIP was used as described in Fig 1.

A new *Δura4 Δpub1* double mutant was constructed to further examine the effect of *pub1* deletion on the suppression of cell lysis. Cell lysis in the *Δura4* cells was significantly suppressed by deletion of the *pub1* gene when cells were grown on YPD medium (Fig 2A). Next, we wished to determine the role of Pub1 in cell lysis. Because strong suppression was observed in uracil-auxotrophic *ura1*, *ura2*, *ura3*, and *ura5* mutants [7], we first asked whether *Δpub1* was also a uracil auxotroph. However, *Δpub1* cells did not display uracil auxotrophy (Fig 2B), indicating that Pub1 was unlikely to be involved in the uracil metabolic pathway. Next, to ask whether Pub1 was involved in uracil uptake, *Δpub1* cells were tested for sensitivity to 5-FU, a toxic uracil analog. When grown on EMM or YPD, *Δpub1* cells exhibited higher sensitivity to 5-FU than wild-type cells or strains carrying other mutations (Fig 2B and 2C). This suggested that the uracil uptake system was up-regulated in *Δpub1* cells. We observed that deletion of the gene encoding Fur4, a uracil transporter, conferred resistance to 5-FU, as previously shown [21] (Fig 2B). We reasoned that Pub1 might affect Fur4, and created a *Δfur4 Δpub1* double deletion mutant to examine this interaction. The high sensitivity of *Δpub1* cells to 5-FU was suppressed by *fur4* deletion (Fig 2B and 2C), which suggested that Pub1 had a role in the uracil transport pathway involving Fur4.

Next, we created the *Δura4 Δfur4* and *Δura4 Δfur4 Δpub1* mutants to assess the effect of *fur4* deletion on cell lysis in a *Δura4* background. As described above, cell lysis was suppressed by *Δpub1*; however, deletion of *fur4* reversed this suppressive effect (Fig 2D). This provided further support for the hypothesis that Pub1 affected the function of the uracil transporter Fur4.

### Fur4 plays a role in the uptake of uracil

**Fig 3:**
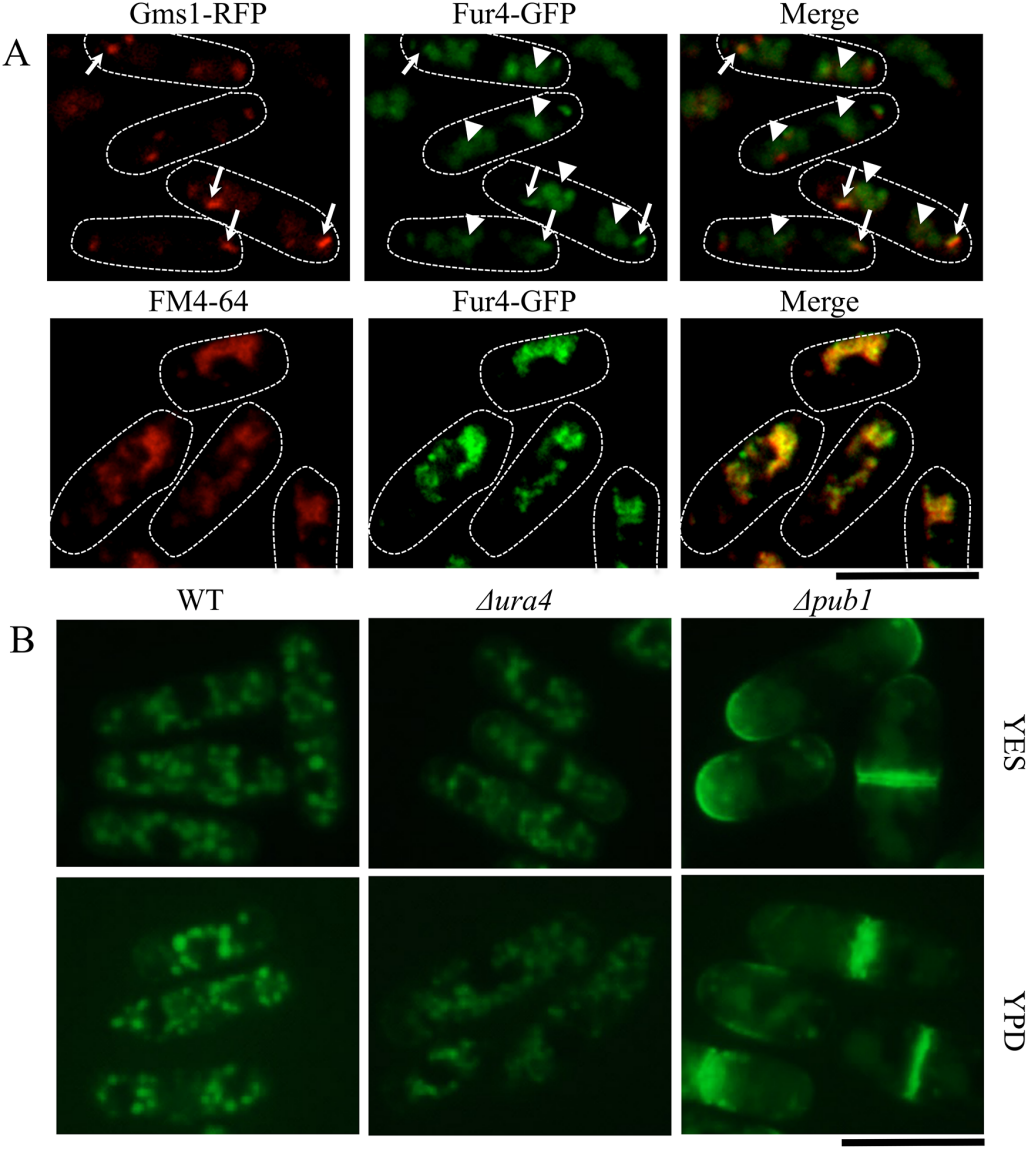
Localization of Fur4-GFP. (A) KNP95 (Δ*ura4 fur4-GFP*) cells carrying pAU-gms1-RFP (*ura4* ^+^) were cultured in EMM medium for 12 h at 30°C and observed using fluorescence microscopy. Arrows show co-localization of Fur4-GFP with Golgi marker and arrow heads show only localization of Fur4-GFP (top panel). KNP74 (*fur4-GFP*) cells were cultured in EMM medium for 12 h at 30°C and shifted to EMM containing 8μM FM4-64. These cells were cultured for 1 h and washed twice with EMM medium to visualize vacuoles and Fur4-GFP (bottom panel). (B) KNP74 (WT *fur4-GFP*), KNP95 (Δ*ura4 fur4-GFP*), and KNP83 (Δ*pub1 fur4-GFP*) cells were grown in YES medium for 12 h. These cells were washed twice with sterile water and suspended in YES or YPD medium and incubated at 30°C for 1 h. These cells were observed using fluorescence microscopy. Bars: 10 μm.

One possible explanation for the mutant data described above is that Pub1 down-regulates Fur4. This might be achieved by altering the localization of Fur4 or, as Pub1 has ubiquitin E3 ligase activity [11], through breakdown of Fur4. A strain expressing a Fur4-GFP fusion protein was constructed to examine the first possibility. The Fur4 protein was tagged with GFP at the C-terminus and chromosomally integrated into the native *fur4* locus to express under the native promoter. The tagged strain was checked for its sensitivity to 5-FU in EMM medium. No significant difference was observed by fusing GFP with *fur4* (S1 Fig). The *Δura4* cells expressing Fur4-GFP carrying pAU-gms1-RFP (*ura4* ^+^) were grown on YES or EMM medium and the localization of Fur4-GFP was observed. Fur4-GFP localized to some subcellular compartments (Fig 3A). Fur4-GFP co-localized with RFP-tagged Gms1 (Arrow), which was known to localize to the Golgi [23] (Fig 3A, top panel). Fur4-GFP also localized to other compartments (Arrow head). The Fur4-GFP exhibited a vacuolar pattern of fluorescence corresponding to the staining pattern of FM4-64 (Fig 3A, bottom panel). Thus, Fur4-GFP localizes to the Golgi and vacuoles in wild type.

Localization of Fur4-GFP was next examined in wild-type, *Δura4* or *Δpub1* cells. When grown in YES or YPD media, Fur4-GFP localized primarily to the plasma membrane in *Δpub1* cells, indicating that Pub1 was important for localization of Fur4 at the Golgi apparatus (Fig 3B).

**Fig 4:**
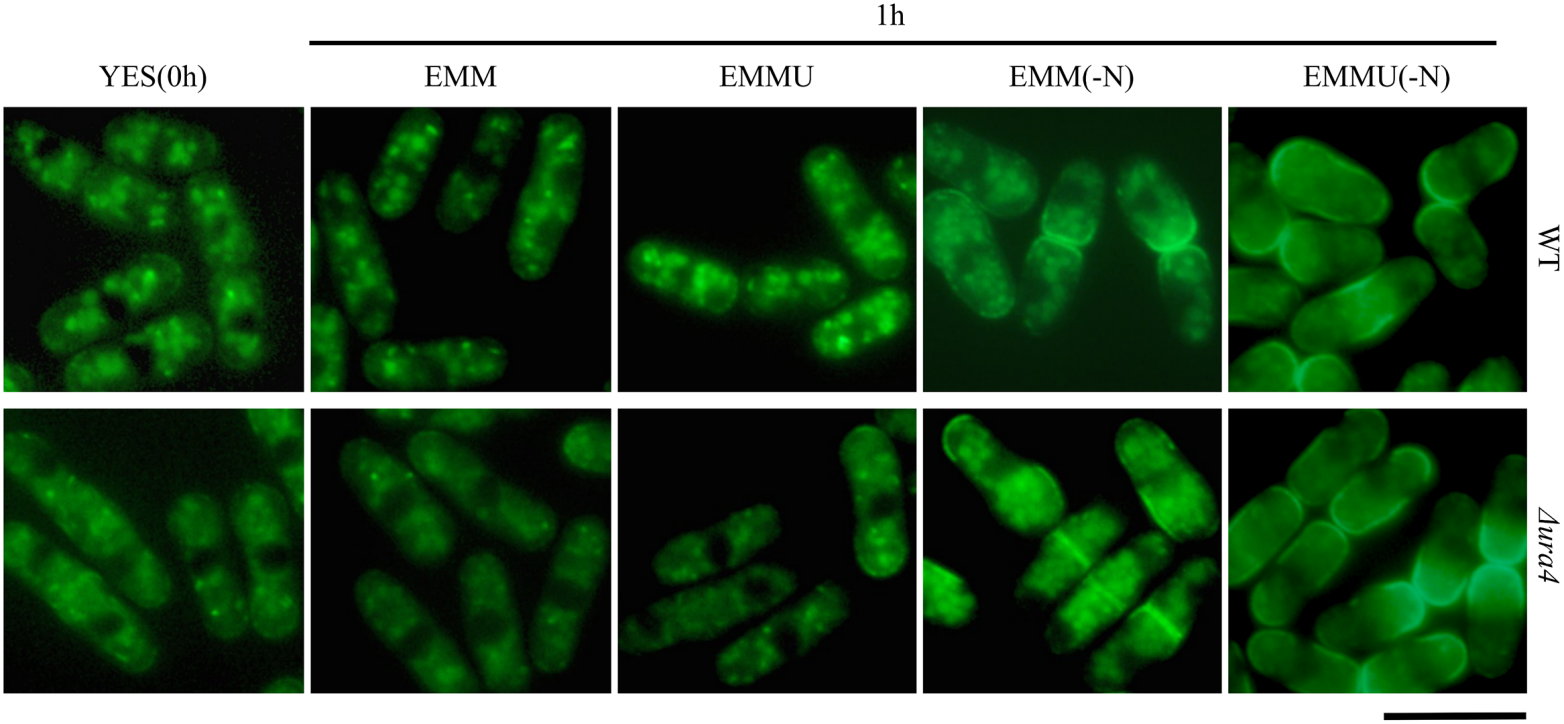
Localization of Fur4-GFP under nitrogen starvation conditions. (A) KNP74(*ura4* ^+^), and KNP95 (Δ*ura4*) cells expressing Fur4-GFP were grown in YES liquid medium for 12 h. Cells were washed twice with sterile water and suspended in EMM, EMMU, EMM(-N), or EMMU(-N) media and incubated at 30°C for 1 h. Cells were observed using fluorescence microscopy. Bar: 10 μm.

In *S*. *cerevisiae*, Fur4p localizes to the membrane upon uracil starvation. To determine whether such regulation occurred in *S*. *pombe*, Fur4 was examined in wild-type and *Δura4* cells, but no apparent difference in localization was observed (Fig 4). Localization of Fur4-GFP was no significant different when cells were cultured in long time (0, 6 or 12h) (S2 Fig). We next examined the localization of Fur4-GFP cells cultured in EMM, EMM(-N), EMMU, and EMMU(-N) medium. In contrast to a slight membrane localization of Fur4-GFP in cells grown in EMM and YES, clear membrane localization of it was observed in both wild-type and *Δura4* cells grown in EMMU(-N) medium (Fig 4). Some membrane localization was also seen in EMM(-N) medium. This demonstrated that nitrogen starvation, but not uracil starvation, induced membrane localization of Fur4 in *S*. *pombe*, indicating that the regulation of Fur4 in *S*. *pombe* differed from that of Fur4p in *S*. *cerevisiae*.

**Fig 5:**
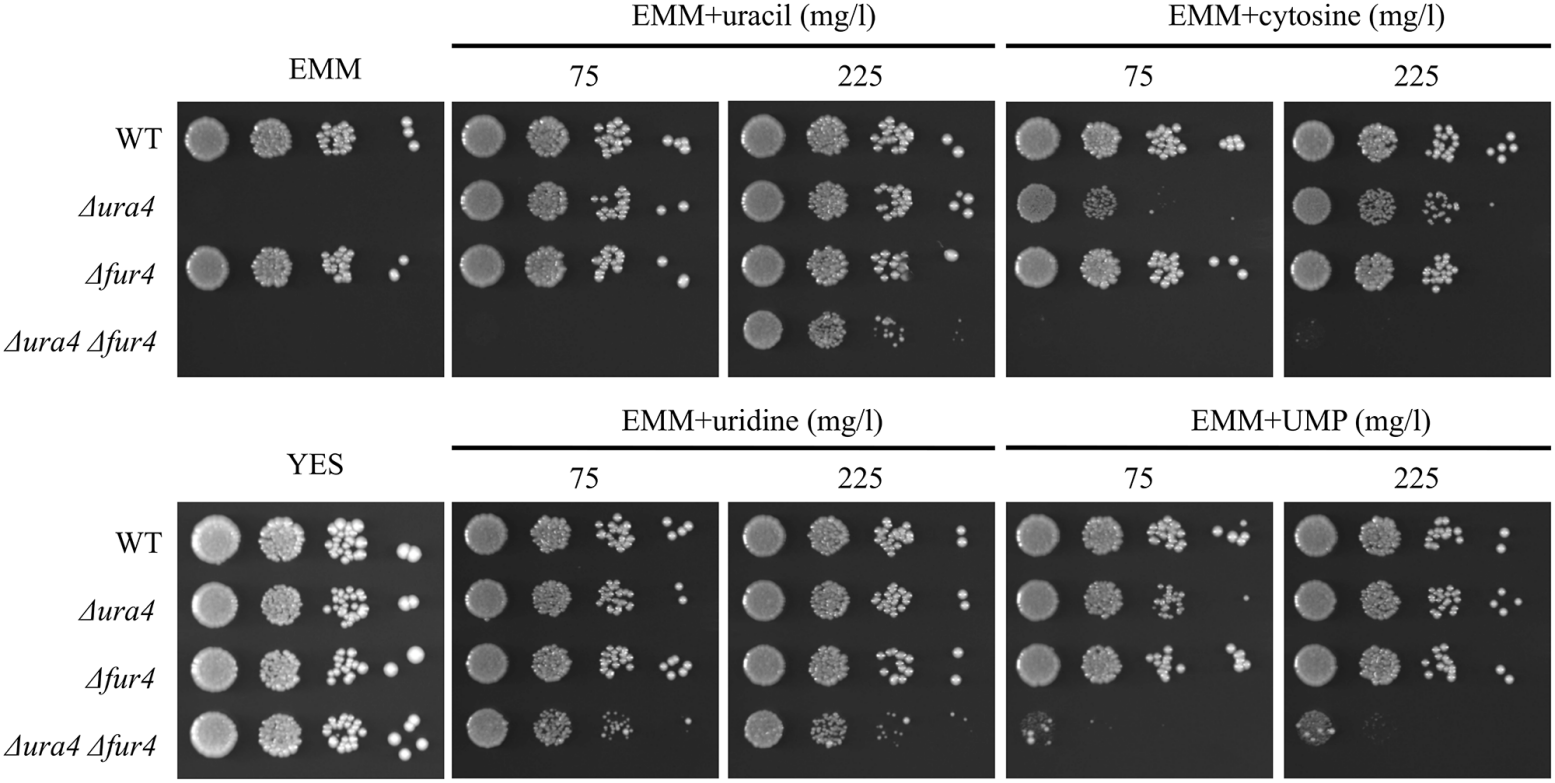
Growth recovery by supplementation of various nucleotide derivatives. L972 (*ura4* ^+^), KNP16 (Δ*fur4*), UMP31 (Δ*ura4*), and KNP27 (Δ*ura4* Δ*fur4*) cells were grown in YES medium for 12 h. Cells were spotted on EMM containing uracil, cytosine, uridine, or UMP (75 or 225 mg/l) and incubated at 30°C for 3 days.

Fur4 acts as a uracil transporter with uracil uptake activity in *S*. *pombe* [24]. However, it was unclear whether, as with *S*. *cerevisiae* Fur4p, *S*. *pombe* Fur4 was a uracil-specific transporter. To examine this, we tested the substrate specificity of the Fur4 transporter. Uracil-auxotrophic strains that lacked *ura4* were unable to grow on EMM minimum medium, but could grow on minimum medium containing 75 mg/l of uracil (Fig 5). Double *Δura4 Δfur4* deletion mutants could not grow on EMMU medium containing 75 mg/l of uracil, but could grow when the amount of uracil was raised to 225 mg/l. This showed that Fur4 was necessary for the efficient utilization of extracellular uracil, but also that Fur4 was not the sole transporter of uracil in *S*. *pombe*. Cytosine, uridine, and UMP were also tested for their impact on growth (Fig 5). The addition of these components supported growth of the Δ*ura4* strain. This was as expected because the salvage metabolic pathway from cytosine to uracil should be functional in this strain. However, while the *Δura4 Δfur4* double mutant was able to grow with uridine supplementation, no growth was seen on medium containing cytosine, and only minimal growth was observed with UMP. These results showed that Fur4 enhanced the utilization of extracellular uracil and UMP, but not uridine, and was absolutely required for the utilization of extracellular cytosine.

### Uracil concentration impacts cell lysis

**Fig 6:**
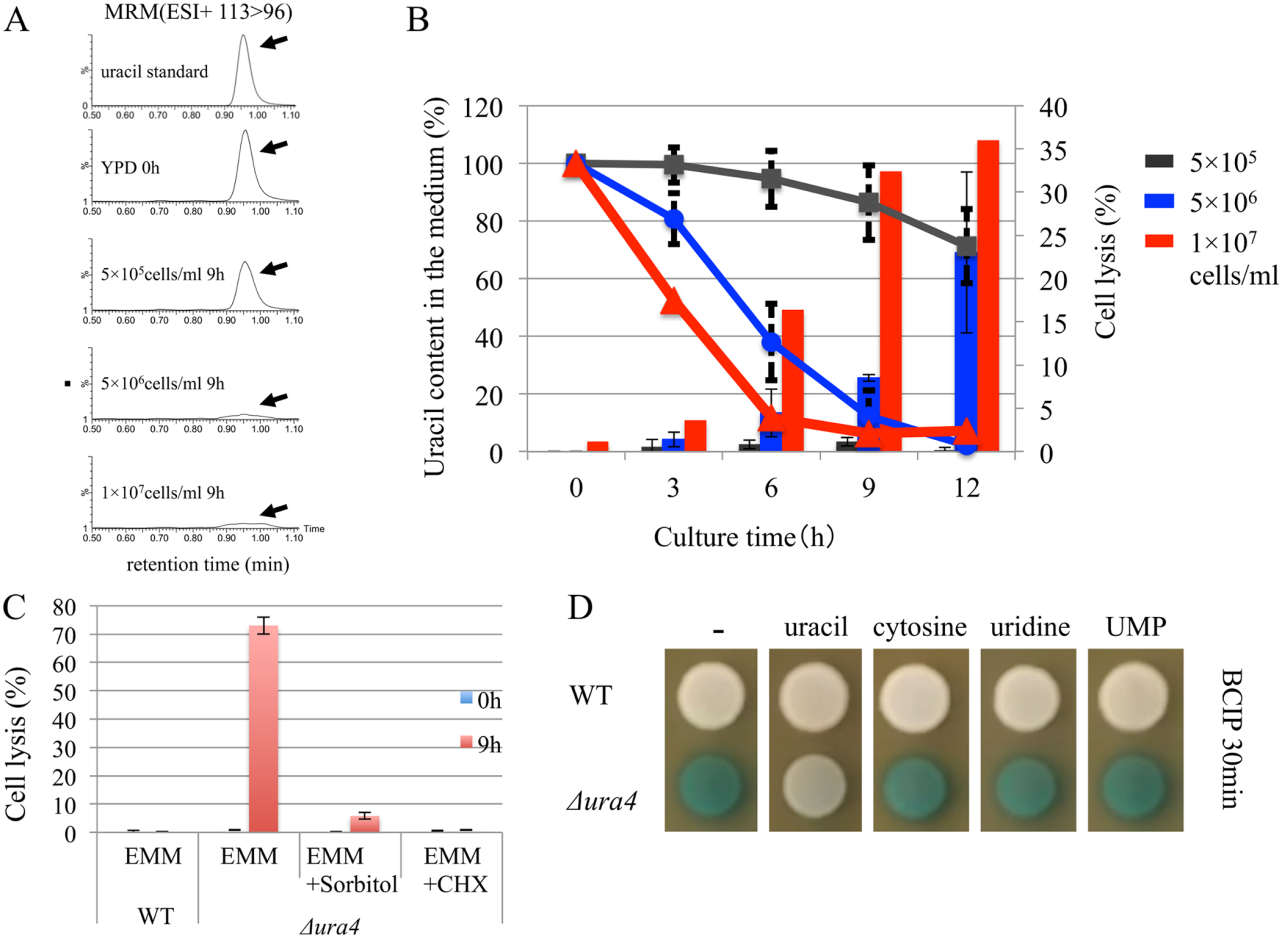
Induction of cell lysis by uracil starvation. (A) UMP31 (Δ*ura4*) cells were grown in YES medium for 12 h. Cells were washed twice with sterile water and then grown in YPD medium at initial cell counts of 5 × 10^5^, 5 × 10^6^, or 1 × 10^7^ cells/ml. The chromatography of LC-MS data (MRM (ESI+ 113>96)) obtained at 0 and 9h are shown as representatives. Arrows show uracil peaks. (B) All data of uracil concentration measured by LC-MS are plotted in the graph. The uracil concentration in the medium (lines) and the extent of cell lysis (bars) were measured after 0, 3, 6, 9, and 12 h of incubation (right). Error bars indicate standard deviation. (C) L972 (*ura4* ^+^) and UMP31 (Δ*ura4*) cells were grown in YES medium for 12 h, washed twice with sterile water, and suspended in EMM medium with or without CHX (100 μg/ml) or 1 M sorbitol and incubated at 30°C. Cell lysis was measured after 0 and 9 h of incubation. Error bars indicate standard deviation. (D) Wild-type and Δ*ura4* cells were grown in YES medium for 12 h. Cells were then spotted on YPD containing uracil, cytosine, uridine, or UMP (each 300 mg/l) and incubated at 30°C for 3 days. For the alkaline phosphatase assay, BCIP was used as described in Fig 1.

Previous research suggested that cell lysis in *Δura4* cells was induced during the stationary phase [7]. The suppression of lysis, even at the stationary phase, by uracil supplementation suggested that uracil starvation was the cause of cell lysis in *Δura4* cells. To test this, we examined induction of cell lysis in three *Δura4* cultures initiated with differing cell numbers (5 × 10^5^, 5 × 10^6^, or 1 × 10^7^ cells/ml). Cell lysis and uracil concentration in the medium were determined. The proportion of lysed cells increased with increasing initial cell concentration, and uracil concentration in the media decreased more rapidly with higher initial cell concentrations (Fig 6A and 6B). Cell lysis was strongly induced when *Δura4* cells were grown in EMM medium, which did not contain uracil (Fig 6C), supporting the supposition that uracil starvation was responsible for the induction of cell lysis. Cell lysis in EMM was also suppressed in the presence of cyclohexidine or 1 M sorbitol (Fig 6C). Sorbitol prevents bursting by maintenance of high osmolarity. Cyclohexidine inhibits protein synthesis, preventing the cellular processes that lead to lysis. Lysis of *Δura4* cells was suppressed by the addition of uracil, but not by supplementation with uridine, UMP, or cytosine (Fig 6D).

### Uracil transport activity is down-regulated in Δ*fur4 strains*

**Fig 7:**
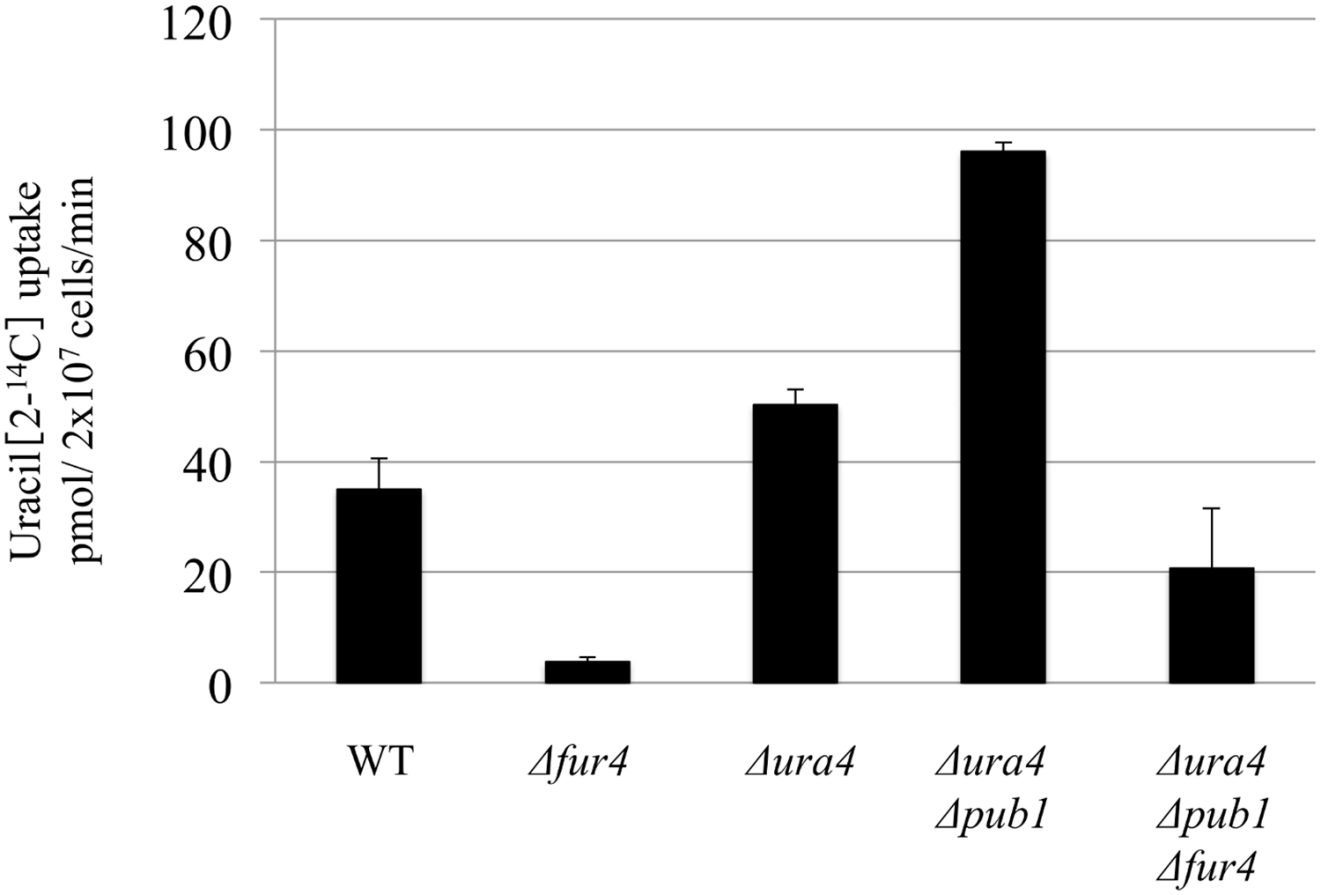
Uracil transport assay. Exponentially growing L972 (WT), KNP16 (Δ*fur4*), UMP31 (*Δura4*), KNP32 (Δ*ura4 Δpub1*), and KNP38 (*Δura4 Δpub1 Δfur4*) cells were grown in YES medium for 12 h. Uracil uptake was monitored using [U-^14^C]uracil. After 1 min, cells were filtered on cellulose acetate membrane filter and washed three times. Radioactivity was measured using a liquid scintillation counter with xylene scintillator. Experiments were performed in triplicate, and error bars indicate standard deviation.

The uracil transporter Fur4 localized predominantly to the membrane in the *Δpub1* strain, which suggested that uracil uptake might be up-regulated in *Δpub1* cells. To test this, we used a [U-^14^C] uracil assay to measure uracil uptake in mutant and wild-type strains (Fig 7). Consistent with previous results [21], only minimal uracil uptake activity was observed in the *Δfur4* strain. Uracil uptake was up-regulated in *Δura4 Δpub1* cells and, to a lesser extent, in *Δura4* cells. Uptake was down-regulated in *Δura4 Δpub1 Δfur4* cells (Fig 7). These results were consistent with the resistance to 5-FU exhibited by strains containing the *fur4* deletion (Fig 2B). Taken together, these data suggest that up-regulation of uracil uptake is dependent on Fur4 and that Pub1 regulates uracil uptake via Fur4.

### Fur4 is ubiquitinated in wild-type and Δ*pub1* cells

**Fig 8:**
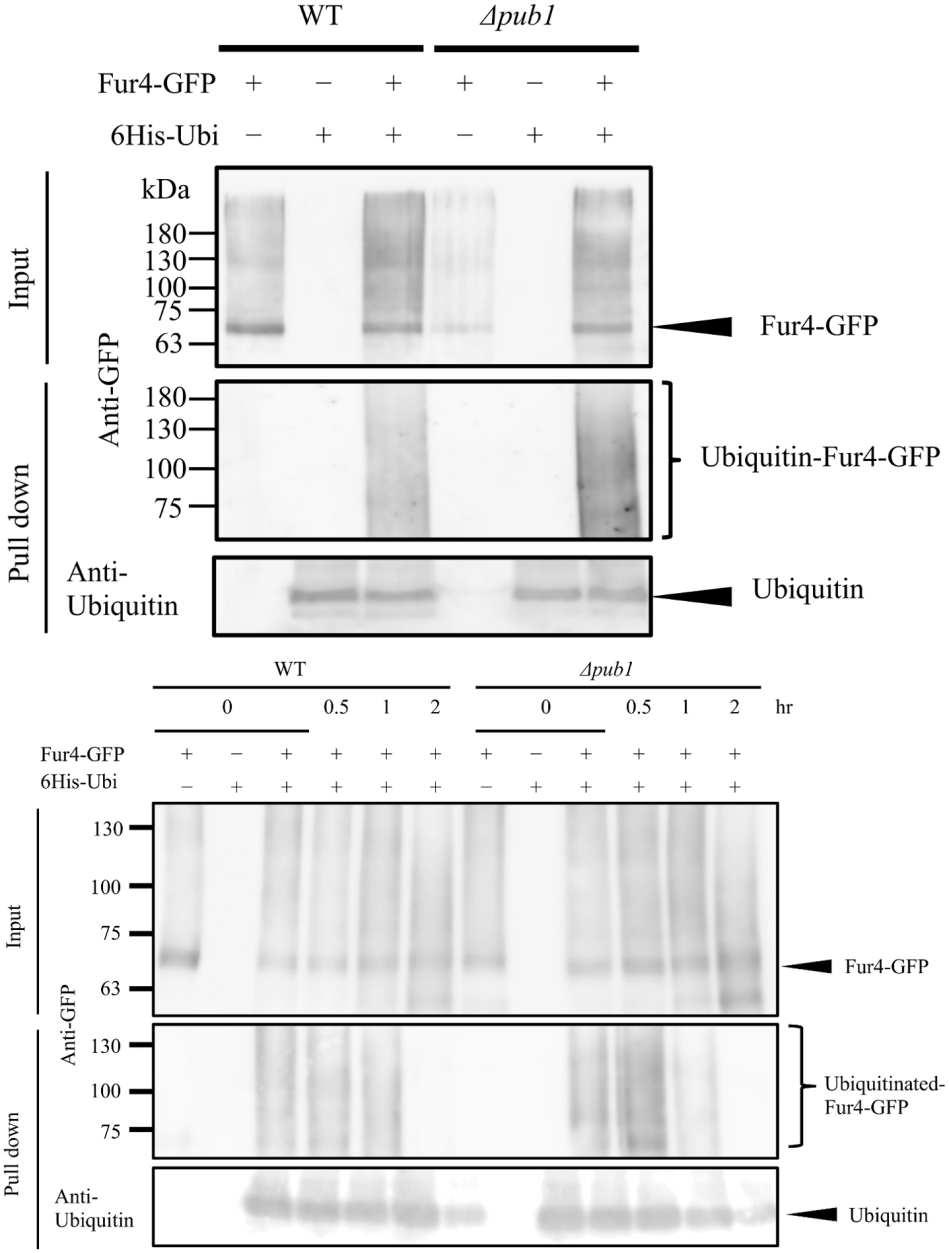
Ubiquitination of Fur4. (A) PR109 (WT) and KNP63 (Δ*pub1*) carrying pREP42(-) or pREP42-Fur4-GFP and carrying pREP1 (-) or pREP1-6His-Ub (+) were grown in EMM. Proteins tagged with 6His-Ubiquitin were purified from protein extracts using Ni^2^-NTA. Immunoblotting was performed with an anti-GFP antibody (upper and middle) and with an anti-ubiquitin antibody as a loading control (bottom). (B) The same transformants were grown in EMM for 16 h. Cells were harvested by centrifugation, washed twice with dH_2_O and further grown in EMM(-N) medium for 2 h. After proteins were extracted and pulled down by Ni^2^-NTA, immunoblotting was performed as in (A).

Pub1, which is an E3 ubiquitin ligase, regulates localization of target proteins in some cases [11]. We reasoned that Pub1 might ubiquitinate Fur4 and thereby regulate its localization. To test this, Fur4-GFP was ectopically expressed in wild-type and Δ*pub1* strains and its ubiquitination was examined. Prior to this experiment, we verified the function of Fur4-GFP does not differ with Fur4 by checking 5-FU sensitivity and localization in Δ*ura4*Δ*fur4* and Δ*pub1* cells (S1A and S1B Fig). A set of plasmids pREP42 (or pREP42-Fur4-GFP) and pREP1 (or pREP1-6His-Ubiquitin) were introduced into wild-type and Δ*pub1* strains. After these transformants were growth in EMM medium, cellular proteins were extracted and detected on western blots using an anti-GFP antibody. Fur4-GFP was observed as smears in wild-type and Δ*pub1* cells (Fig 8). Extracts were then incubated with Ni^2^-NTA agarose beads to purify 6His tagged ubiquitin conjugated proteins. Purified extracts were detected on western blots with anti-GFP or anti-ubiquitin (control) antibodies (Fig 8A). Ni^2^-NTA-purified Fur4 was ubiquitinated in both wild-type and Δ*pub1* cells, which suggested that Pub1 was not directly involved in ubiquitination of Fur4.

We next examined ubiquitination of Fur4-GFP during cells were incubated under nitrogen starved condition, since we observed membrane localization of Fur4-GFP increased by nitrogen starvation (Fig 4). We cultured cells harboring pREP42-Fur4-GFP and pREP1-6His-ubiquitin in EMM medium and further incubated in EMM (-N) medium for 2 h. After confirming intensive membrane localization of Fur4-GFP as in Fig 4, proteins were extracted. The proteins were detected on western blot using anti-GFP and anti-ubiquitin antibodies (Fig 8B). Extracts were then incubated with Ni^2^-NTA agarose beads to purify 6His tagged ubiquitin conjugated proteins. In this pull down experiment, ubiquitination of Fur4-GFP was detected before cells were incubated without nitrogen, but it gradually disappeared during nitrogen starvation. After 2 h nitrogen starvation, ubiquitinated Fur4-GFP was not detected in the pulled down samples both in wild type and Δ*pub1* deletion background.

## Discussion

### Cell lysis in *ura4* mutants is suppressed in the absence of *pub1*

In this study, we explored the mechanisms by which cell lysis was induced in *S*. *pombe ura4* mutants through the analysis of suppressors obtained from screening a *S*. *pombe* 3,400 non-essential gene deletion library. Several suppressors were identified. Deletion of the *pub1* gene, which encoded E3 ubiquitin ligase, produced a particularly strong suppression phenotype (Fig 1A). Previously identified suppressors were either directly involved in *de novo* UMP synthesis, such as mutations in *ura1*, *ura2*, *ura3*, or *ura5*, or were indirectly involved in UMP synthesis, such as mutations affecting synthesis of CoQ (a cofactor of the Ura3 reaction) [7,25]. However, *pub1* deletion did not render uracil auxotrophy, and the *Δpub1* strain had high 5-FU sensitivity, indicating that the mechanism of suppression was not related to a *de novo* UMP pathway. Cell lysis was suppressed by supplementation of uracil in YPD, suggesting that Pub1 might be involved in uracil uptake. The involvement of Pub1 in uracil uptake was confirmed by its alteration of the localization of the uracil transporter Fur4, which was identified in a previous study [21,24].

Genetic experiments consistently supported the relationship between Pub1 and Fur4. High sensitivity to 5-FU in the Δ*pub1* strain was suppressed by deletion of *fur4*. Furthermore, the Δ*ura4* Δ*pub1* Δ*fur4* strain, but not the Δ*ura4* Δ*pub1* strain, underwent lysis when grown on YPD medium (Fig 2). Fur4 predominantly localized to the Golgi apparatus and vacuoles under normal growth conditions but accumulated at the cell membrane in the *pub1* mutant, underlining the importance of Pub1 for Fur4 localization (Fig 3). The high sensitivity to 5-FU and the suppression of cell lysis observed in the Δ*pub1* strain could be explained by the accumulation of Fur4 at the membrane. Consistent with Fur4 membrane localization in Δ*pub1*, uracil uptake was higher in the *pub1* mutant than in the wild type (Fig 7). Thus, the identification of *pub1* in the initial suppressor screen could be explained by the increased uptake of uracil in Δ*pub1* mutants as a result of Fur4 localization at the membrane.

**Fig 9:**
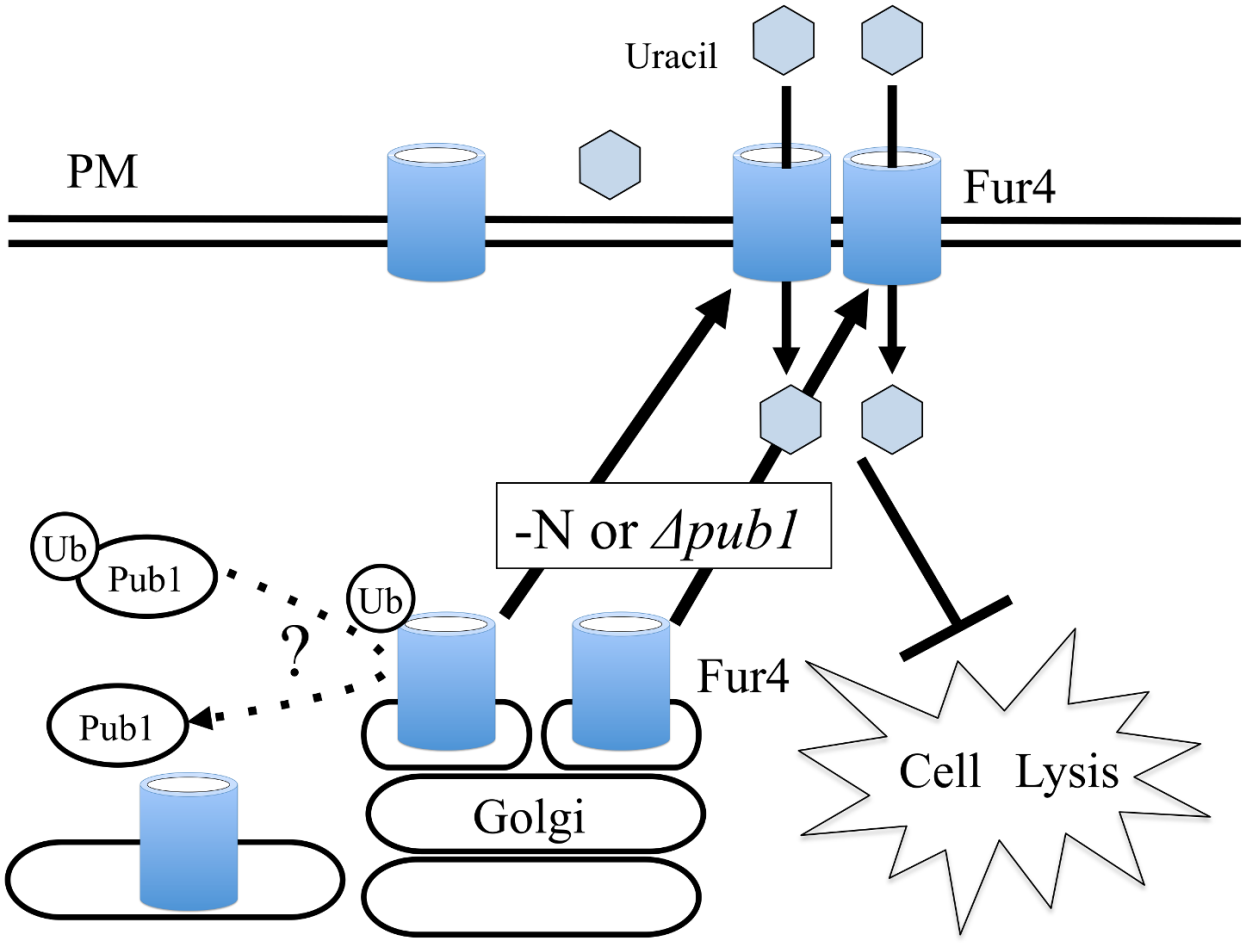
Proposed model for interaction of Pub1 and Fur4. The uracil transporter Fur4 localizes to Golgi or vacuoles under normal growth conditions and shifts to the plasma membrane when nitrogen is consumed. Fur4 is also localized to the plasma membrane when the *pub1* gene is deleted. Increased uracil uptake in *pub1* mutants by the accumulated Fur4 localization in the membrane rescues the cell lysis phenotype. Ubiquitination of Fur4 is observed, but the mechanisms underlying Pub1 regulation of Fur4 localization remains to be determined. PM, Plasma Membrane.

A diagram outlying the proposed interactions of Fur4 and Pub1 is given in Fig 9. This cartoon is supported by our experimental observations, but at least two major questions relating to this mechanism remain. First, why does supplementation with uracil suppress cell lysis, and second, how does Pub1 affect Fur4 localization?

We used a supplementation approach to address the first question. Uracil, but not cytosine, uridine, or UMP, was able to suppress cell lysis (Fig 5); however, all four supplements rescued the inability of *ura4* mutants to grow on minimal medium (Fig 5). These results suggested that uracil had a specific role in the suppression of cell lysis and that the suppression did not involve enforcement of the UMP synthesis salvage pathway. Previous experiments showed that OMP, a precursor of the Ura4 reaction, accumulated when Δ*ura4* cells were grown in YPD medium and that addition of uracil decreased OMP accumulation [7]. It was therefore possible that OMP acted as a key component in the cell lysis pathway. Uracil is converted to UMP by condensation with 5-phosphoribosyl 1-pyrophosphate (PRPP). This suggests that supplementation with uracil may provide a competitor for the Ura5 reaction (condensation of PRPP with orotate), thereby, as we observed, decreasing levels of OMP. Cytosine, uridine, and UMP are unlikely to compete with the Ura5 reaction. However, this competition hypothesis remains to be proved.

To address the second question, we asked whether, given that *pub1* encodes E3 ubiquitin ligase, variable ubiquitination might explain the effect of Pub1 on Fur4 localization. However, Fur4 ubiquitination did not appear to differ between wild-type and Δ*pub1* strains (Fig 8A). This suggested that the E3 ubiquitin ligase activity of Pub1 did not act directly on Fur4. Similarly, a GPI anchored protein, Ecm33, whose localization is also regulated by Pub1, exhibits no clear change in ubiquitination pattern in the absence of Pub1 [14]. Conversely, the amino acid transporter Aat1 is ubiquitinated by Pub1, and this ubiquitination regulates Aat1 localization [11]. Our observations led to the hypothesis that Fur4 might have multiple mono-ubiquitination sites that play distinct roles in its regulation. Such subtle modification differences would not have been discernible in our western analysis. Alternatively, Pub1 may act on a separate target that then regulates Fur4. Multiple ubiquitinations were proposed to play a role in the activity of the amino acid transporter Cat1 [12], and the ubiquitination of adaptor protein Any1 was involved in the regulation of amino acid transporter Aat1 and Cat1 [11,12]. However, the question of how Fur4 is regulated by Pub1 remains to be answered.

### Mechanisms of uracil uptake differ between *S*. *cerevisiae* and *S*. *pombe*

The regulation of the *S*. *cerevisiae* uracil transporter Fur4p has been elucidated [26–28]. Degradation of Fur4p is induced by ubiquitination of two lysine residues (Lys38 and 41) by Rsp5p, a homolog of *S*. *pombe* Pub1 [29,30]. The ubiquitinated Fur4p is rapidly internalized and degraded in a multivesicular body (MVB) pathway [28]. When cells are grown on medium containing uracil, Fur4p is internalized and degraded [29,30]. This regulatory pathway can also be induced by stressors such as heat or H_2_O_2_ [30].

In *S*. *pombe*, down-regulation of Fur4 was not induced by uracil (Fig 3). We also observed that, when wild-type and Δ*ura4* strains were grown under nitrogen starvation conditions, Fur4 localized to the cell surface and septum (Fig 4). No similar observations were seen with *S*. *cerevisiae* Fur4p [31]. Intriguingly, ubiquitined Fur4 was not detected in the pull down experiment of the proteins taken from the cells grown under nitrogen starved condition for 2 h (Fig 8B). But ubiquitination of Fur4 did not disappear in *pub1* deletion mutants without starvation. Our result indicates that nitrogen starvation is critical for de-ubiquitination of Fur4-GFP. We also noticed a faster de-ubiquitination of Fur4-GFP in *pub1* deletion mutants than wild type under 1h nitrogen starved condition (Fig 8B). We think that the balancing of ubiquitination (possibly mediated by Pub1) and de-ubiquitination of Fur4-GFP through nitrogen starvation is operating in this regulation. Further analysis is necessary to understand this regulatory mechanism of Fur4 by ubiqutination.

In addition, Fur4p is a high-affinity uracil transporter that does not transport cytosine [32]. Genetic data indicated that *S*. *pombe* Fur4 was involved in transport of uracil, cytosine, and UMP (Fig 5). Collectively, these data indicate that the regulation of uracil uptake by Fur4 and Fur4p differs substantially between *S*. *pombe* and *S*. *cerevisiae*.

### Uracil concentration is critical for cell lysis in Δ*ura4* cells

Our experiments demonstrated that the cellular uracil concentration was critical for cell lysis in the Δ*ura4* strain (Fig 6A and 6C). The initial finding that cell lysis was induced when *ura4* mutants were grown in YPD medium was complemented by the observation that cell lysis was induced immediately upon cultivation of Δ*ura4* cells in EMM medium (Fig 6A). The time of uracil consumption in YPD coincided with the time of cell lysis (Fig 6B). Lysis of Δ*ura4* cells is strongly related to the level of uracil concentration. Our current hypothesis is that polypeptone within the media contains some factor(s) that facilitates uracil consumption, but this remains to be proved.

In conclusion, cell lysis in *S*. *pombe* Δ*ura4* cells is triggered by cellular uracil depletion and consequent OMP accumulation. OMP then likely weakens the cell wall structure by an unknown mechanism, allowing cells to be easily broken.

## Supporting Information

S1 Fig Fur4-GFP retains the function indistinguishable with Fur4. (A) L972 (WT), KNP16 (Δ*fur4*), KNP74 (Fur4-GFP) and KNP27 (Δ*ura4* Δ*fur4*) carrying pREP42 or pREP42-Fur4-GFP (#1 and #2 only differ in an isolated transformant) were grown on YES or EMM plate for 24 h. The indicated cells were streaked on EMM in the absence or the presence of 5-FU (50 μM) and incubated at 30°C for 3 days. (B) PR109 (WT) and KNP63 (Δ*pub1*) carrying pREP42 or pREP42-Fur4-GFP cells were grown in EMM+thiamine liquid medium for 12 h. Cells were washed twice with sterile water and suspended in EMM and incubated at 30°C for 18 h. Cells were observed by fluorescence microscopy. Bar: 10μm.
(TIFF)

S2 Fig Localization of Fur4-GFP does not differ by long time cultivation. KNP74 (Fur4-GFP) was grown in YES liquid medium for 12 h. Cells were washed twice with sterile water and suspended in EMM media and incubated at 30°C for 0, 6, and 12 h. Cells were observed by fluorescence microscopy. Bar: 10μm.
(TIFF)

S1 Table Primers used in this study. (DOCX)
