# Supplementary figures and images for: Cell Lysis in S. pombe ura4 Mutants Is Suppressed by Loss of Functional Pub1, Which Regulates the Uracil Transporter Fur4
Source: PLoS One. 2015 Nov 4;10(11):e0141796. doi: 10.1371/journal.pone.0141796 (PMC4633276; doi:10.1371/journal.pone.0141796)

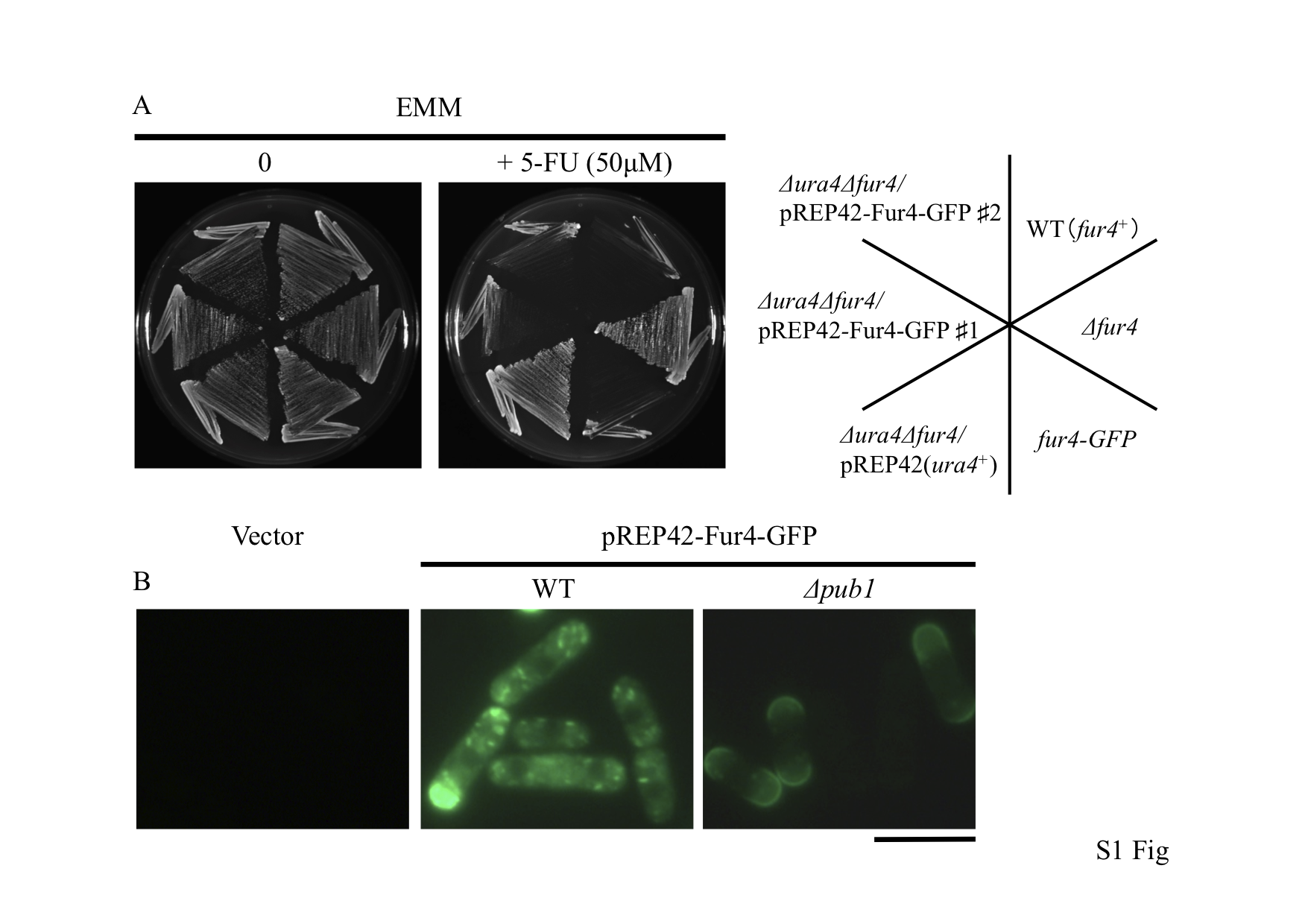

Supplement: S1 Fig — (A) L972 (WT), KNP16 (Δfur4), KNP74 (Fur4-GFP) and KNP27 (Δura4 Δfur4) carrying pREP42 or pREP42-Fur4-GFP (#1 and #2 only differ in an isolated transformant) were grown on YES or EMM plate for 24 h. The indicated cells were streaked on EMM in the absence or the presence of 5-FU (50 μM) and incubated at 30°C for 3 days. (B) PR109 (WT) and KNP63 (Δpub1) carrying pREP42 or pREP42-Fur4-GFP cells were grown in EMM+thiamine liquid medium for 12 h. Cells were washed twice with sterile water and suspended in EMM and incubated at 30°C for 18 h. Cells were observed by fluorescence microscopy. Bar: 10μm. (TIFF) [file pone.0141796.s001.tiff]

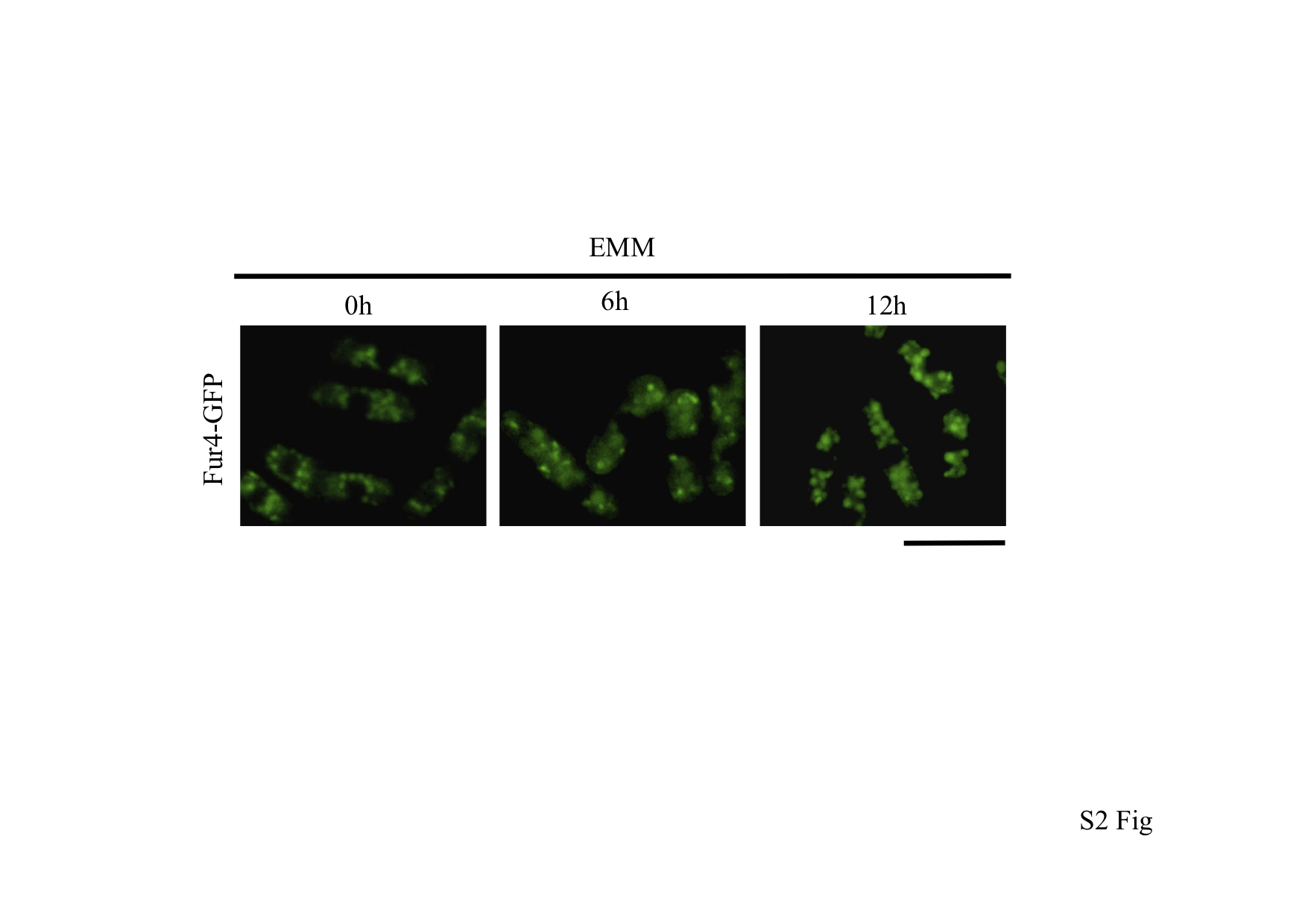

Supplement: S2 Fig — KNP74 (Fur4-GFP) was grown in YES liquid medium for 12 h. Cells were washed twice with sterile water and suspended in EMM media and incubated at 30°C for 0, 6, and 12 h. Cells were observed by fluorescence microscopy. Bar: 10μm. (TIFF) [file pone.0141796.s002.tiff]
