# Supplementary material for: Cell Lysis in S. pombe ura4 Mutants Is Suppressed by Loss of Functional Pub1, Which Regulates the Uracil Transporter Fur4
Source: PLoS One. 2015 Nov 4;10(11):e0141796. doi: 10.1371/journal.pone.0141796 (PMC4633276; doi:10.1371/journal.pone.0141796)
Supplement: S1 Table — (DOCX) [file pone.0141796.s003.docx]

S1 Table: Primers used in this study

| Primer | Sequence |
| --- | --- |
| fur4-d-A | 5′-CATTGAGGGAATTGAAAGGCG-3′ |
| fur4-d-B | 5′-GGGGATCCGTCGACCTGCAGCGTACGACAAGACTTTTGTAGGGAAAG-3′ |
| fur4-d-C | 5′-GTTTAAACGAGCTCGAATTCATCGATGGTTTGATCTCGTCTTAATAG-3′ |
| fur4-d-D | 5′-CTGTGTTCAATAAGGCGT-3′ |
| fur4-d-chk1 | 5′-TTCCCGAAGAAGCTGATGAC-3′ |
| fur4-d-chk2 | 5′-CAAGTCCATACCGTGACATTC-3′ |
| fur4-tag-A | 5′-GCAGCCCCTATTACTTTTGG-3′ |
| fur4-tag-B | 5′-GGGGATCCGTCGACCTGCAGCGTACGAAGGAAAACAACCCGATAATTTTTTGC-3′ |
| fur4-tag-C | 5′-GTTTAAACGAGCTCGAATTCATCGATCATCAAATGGAAATAACACGCC-3′ |
| fur4-tag-D | 5′- CCGCTACTTTGGCACTTG-3′ |
| fur4-tag-chk1 | 5′-GCGTTTAGACAGTAAGCG-3′ |
| fur4-tag-chk2 | 5′-TTACAGCGTCTATTGATCCCG-3′ |
| pub1-d-A | 5′-CGTATTTTCTGTACGCC-3′ |
| pub1-d-B | 5′-GGGGATCCGTCGACCTGCAGCGTACGAGGTAAAATTTCAAACCACGC-3′ |
| pub1-d-C | 5′-GTTTAAACGAGCTCGAATTCATCGATGGATAGCTAGCTATTGATTAC-3′ |
| pub1-d-D | 5′-CACATGTATTGAAGCGGCTATTG-3′ |
| pub1-d-chk1 | 5′-GTCCATAGCTGTTGAAGAG-3′ |
| pub1-d-chk2 | 5′-TCATAGCTCTGCGGTCTG-3′ |
| fur4 P-NdeI | 5'-ATTCATATGATGGAGTCTGTGGATAATAATTC-3' |
| pFA6a-d-SalISmaIF | 5’-CGCTGCAGGTCGCCGGATCCCAGGGTTAATTAAC-3’ |
| pFA6a-d-SalISmaIR | 5’-GTTAATTAACCCTGGGATCCGGCGACCTGCAGCG-3’ |
| 13MYC-SR | 5’-ACACCCGGGAGATCTATATTACCCTGTTA-3’ |
